# Faculty development programs for medical teachers in India

**Published:** 2016-04

**Authors:** SANJAY ZODPEY, ANJALI SHARMA, QUAZI SYED ZAHIRUDDIN, ABHAY GAIDHANE, SUNANDA SHRIKHANDE

**Affiliations:** 1 Public Health Education, Public Health Foundation of India, New Delhi, India;; 2 Community Medicine Department, J N Medical College, Datta Meghe Institute of Medical Sciences, Wardha, Maharashtra, India;; 3 Department of Microbiology, Indira Gandhi Government Medical College, Nagpur, Maharashtra, India

**Keywords:** Faculty, Development, Pedagogy, Medical education

## Abstract

**Introduction:**

India has the highest number of medical colleges in the world and subsequently the higher number of medical teachers. There is a dire need of adopting a systematic approach to faculty development to enhance quality education to meet health challenges for 21st Century. This manuscript provides a landscape of faculty development programs in India, identifying gaps and opportunities for reforms in faculty development.

**Methods:**

Conventionally, FDPs are organized by medical colleges and universities through Basic Courses and Advanced Courses focusing on pedagogy. Medical Council of India is facilitating FDPs through 18 selected regional centers to enable medical teachers to avail modern education technology for teaching from July 2009. Foundation for Advancement of International Medical Education and Research has three Regional Institutes in India.

**Results:**

Recommendations include the need for formulating a national strategy for faculty development to not only enhance the quantity of medical teachers but also the quality of medical education; providing support for Departments of Medical Education/Regional Centers in terms of finance and staffing and incorporation of teaching skills in postgraduate training.

**Conclusion:**

Distance learning courses focusing on educational leadership and pedagogy for medical teachers can be an option to reach a wider audience. FDPs can be an asset in recruiting and retaining teachers as they offer valued professional development opportunities.

## Introduction

“No bubble is so iridescent or floats longer than that blown by the successful teacher”– As stated by the revered physician and medical educator, Sir William Osler, this thought is particularly apposite to the medical education as to other professions. The medical education system in India is the largest in the world consisting of 387 medical colleges with an intake capacity of 51979 students each year (1).^. ^The unprecedented growth of medical institutions in India in the past two decades, almost doubling in strength, has led to a shortage of teachers and created a quality challenge for medical education(2). It is also widely discussed that academic medicine is growing rapidly and struggling to attract younger medical professionals into the educational arm of the profession. Another concern is the lack of formal policy for faculty development. Where the elementary, primary and secondary school teachers have to undergo training in formal schools or colleges of education to be eligible for appointment and promotion, there is no such requirement for appointment of teachers in medical colleges in India(3).

Lancet Commission Report on ‘Health Professionals for a New Century: Transforming Education to strengthen Health Systems in an Interdependent World’ has created a new thrust among the concerned institutions and agencies working in faculty development (4). Recent World Health Organization (WHO) guidelines on ‘Transforming and scaling up health professionals’ education and training’ also emphasized that faculty development will help to address quality and relevance and prepare competent and motivated doctors for the 21^st^ century(5).

As specified by the Medical Council of India (MCI), Faculty Development Programs aim to improve the quality of medical education by training and sensitizing teachers about new concepts in teaching and assessment methods; develop knowledge and clinical skills required for performing the role of competent and effective teachers, administrators, researchers and mentors; assist clinicians to acquire competency in communication and behavioral skills and update knowledge using modern information and research methodology tools(6). However, for the purpose of this manuscript, the thrust is on faculty development mainly focusing on development of the pedagogical skills of the medical teachers. The objective of this report is to provide the historical evolution and growth of FDPs in India and suggest appropriate ways to improve quality of medical education through faculty development.

## Methods

Conventionally, the FDPs in India have been organized by medical colleges and universities through Basic Courses and Advanced Courses on pedagogy envisaged to improve the quality of medical education by training the medical teachers. The purpose of the Basic Courses in Medical Education Technologies (MET) is to provide the basic knowledge, skills and eventually change the attitude of the faculty in medical colleges which the faculty can implement in their day to day practice in different areas of teaching and assessment (classroom, laboratory, clinical and field work). MCI, in 2014, started Advanced Courses in medical education with the aim to develop educational practitioners who can lead informative and instructional and educational changes in their institutions and thereby making the medical education responsive to the health needs of the society. 

Various National Health Committees have played a pivotal role in providing recommendations**. **Bhore Committee recognized the need for training of medical teachers in as early as 1946 and made recommendations for major changes in medical education which included three months of training in preventive and social medicine to prepare “social physicians”. Fifteen years later, Mudaliar Committee in 1961, re-emphasized the need for the “social physician”. Patel Report, in 1971, described a “basic doctor” of modern medicine who would be central to the delivery of primary healthcare and trained through a five-and-a-half years of university education. In 1974, Srivastav Committee advocated the set up for establishment of Medical and Health Education Commission for planning and implementing the reforms needed in health and medical education on the lines of University Grants Commission. An “Expert Committee for Health Manpower Planning, Production and Management” established in 1985 and known as Bajaj Committee, further urged for a formulation of national medical & health education policy (7).

## Results

In 1969, WHO established the International Regional Teacher Training Centers (IRTTC) and the nodal agency was University of Illinois, United States. The IRTTC trained faculty from six Regional Teacher Training Centers (RTTCs). Two RTTCs were established in South-East Asia, one in Sri Lanka and one in Thailand supported by WHO. Government of India, constituted a Working Group on Continued Medical Education in 1974 which recommended National Teacher Training Centre (NTTC). The first NTTC was established in 1975 at JIPMER, Pondicherry and offered the National Courses on Educational Science for Teachers of Health Professions that are held twice a year. In March 2014, NTTC, JIPMER, Pondicherry organized its 69^th^ National Course on Educational Science for Teachers of Health Professions. The Centre also publishes a bi-yearly journal - NTTC Bulletin on Medical Education. Encouraged by the activities of the NTTC at JIPMER, Pondicherry, Ministry of Health and Family Welfare, Government of India established three more centers, at Postgraduate Institute of Medical Education and Research, Chandigarh, Institute of Medical Sciences, Banaras Hindu University, Varanasi, and Maulana Azad Medical College, New Delhi (8, 9). The National Conference on ‘Training Teachers Today for Tomorrow's Needs’ held under the auspices of MCI in September 1994 and also the workshop on ‘Medical Education - An Appraisal’ held under the auspices of MCI in May 1996 have made recommendations for the establishment of Medical Education Unit in every medical college (10).

Consortium of Medical Institutions for Reform of Medical Education also played a crucial role in advancing the FDPs in India from 1989 to 1995. Four medical institutes, viz, All India Institute of Medical Sciences, New Delhi; Christian Medical College, Vellore; JIPMER, Pondicherry and IMS-BHU, Varanasi and the Department of Medical Education, College of Medicine at Chicago, University of Illinois, formed a consortium. Later this consortium was expanded to 16 colleges. The consortium contributed to curriculum development, built consensus and classified essential skills, into ''must know'' and ''good to know'' categories(3).

K.L. Wig Centre for Medical Education and Technology was another strong catalyst in promoting faculty development in India. It was established at All India Institute of Medical Sciences, New Delhi in 1989-90. Rajiv Gandhi University of Health Sciences, Karnataka, Bangalore and The Tamil Nadu Dr. M.G.R. Medical University has a separate department of curriculum development and promotes Health Professionals' Education and aims to develop need based curricula for the medical and paramedical education. The Indian Journal of Medical Education was launched as an official publication of the Indian Association for the Advancement of Medical Education. The Department of Medical Education of K.M.C. Manipal was established in 1985 to bring about improvements in medical education and health care and regularly holds Teaching-Learning Workshop and Student Evaluation Workshops. The National Knowledge Commission was established by the Government of India in 2005 to recommend and undertake reforms in order to make India a knowledge-based economy and society. An important constituent of the National Knowledge Commission functions was professional education, particularly education in the field of medical sciences (11).

MCI has taken a major initiative of conducting FDPs in India by making regulations on Graduate Medical Education in 1997 which made it mandatory for all medical colleges to establish Department of Medical Education. MCI has also selected 18 Nodal Centers, since July 2009. These centers are located at institutions which have trained manpower in Medical Education Technologies (MET) (6). All these nodal centers are engaged in training medical teachers at all the levels including professors and teacher administrators in MCI recommended Basic Courses. This workshop has been made compulsory for the medical teachers and is considered for the credit hours. The Board of Governors of MCI in 2003 approved the recommendations of the Academic Council that faculty should complete training in MCI Basic Courses in MET either, before joining service, after selection or during the probation period. These centers would conduct four Basic Courses i.e., two per academic sessions. One of these workshops would be for coordinators/in-charge of medical education units. Faculty in-charge of these medical education units would be the participants who would attend the Basic Courses in MET at Nodal Centers. In addition, five more faculty members from each medical college would be trained to create a sufficient resource pool.

More than 40000 faculty are estimated to be working in 387 medical colleges based on the MCI norms and 12141 teachers have been oriented by MCI under FDPs through 429 workshops. The Nodal Centers of MCI have trained 5946 medical teachers in 186 workshops since its inception. And through the Medical Education Unit/ Department, 243 workshops have been conducted and 6195 medical teachers have been trained(6). In Academic Council Meeting of MCI held in February 2012, it was decided that within five years, the medical education units in all medical colleges should have all existing faculty trained in Basic Courses and shall conduct the Basic Courses for newly inducted faculty twice a year.

Advanced Course program in MET was approved by MCI in 2010. There are now 42 trained national faculty in India for conducting Advanced Courses. In 2014, MCI launched the Advanced Course in MET in 10 nodal centers across the country (6). In the beginning phases of the conduction of Advance Course, each Nodal Center should have at least 5 faculty having minimal requirements and criteria for resource faculty to conduct the Advanced Course. The course is structured for a period of one year with 2 contact sessions of 5 days (1st contact session 3 days and 2nd contact session 2 days) at the Nodal Center in September and March. The intersession includes online discussions and project submission (12).

The Foundation for Advancement of International Medical Education and Research (FAIMER) is also assisting faculty development in India. The FAIMER Institute, started in 2001, is a two year fellowship focused on educational leadership and methodology. There are seven Regional Institutes in the world out of which three are in India, one in Brazil, one in Southern Africa, one in China and one in Colombia. In India, the first regional institute, Seth G S Medical College was started in July 2005 in Mumbai, India, The second CMCL-FAIMER Regional Institute, fellowship started in January 2006 based at the Christian Medical College at Ludhiana, Punjab. It is based at PSG Institute of Medical Sciences and Research in Coimbatore in southern India.  These three Regional Institutes are open to South Asia health profession educators and sixteen fellows are accepted each year(13). The program is designed to teach education methods, management, and leadership skills, as well as to develop strong professional bonds with other health profession educators around the world.

Various training programs incorporating innovative models for faculty development are being conducted by various academies, associations, consortia and the deemed universities like Indian Association for Advancement of Medical Education, Consortium of Medical Institutions for Reform in Medical Education, Health Science University Initiatives, Consortium of Health Science Universities and Indian Academy of Health Profession Education. Apart from the MCI Nodal Centers, many other health sciences universities have initiated courses and programs in FDPs. Maharashtra University of Health Sciences (MUHS), Nasik, Maharashtra has established the Institute of Medical Education Technology & Teachers’ Training and has completed 12 trainings on “Advanced Certificate Course in Health Sciences Education Technology” till January 2014. It aims to impart advanced educational skills to teachers in positions of academic responsibility in their own institutions. This certificate course is of six months duration in which there is a seven days contact session consisting of full day sessions followed by a six month educational innovation project(14). MUHS publishes three issues yearly of a peer-reviewed, MEDLINE-indexed journal “*Education for Health*” of The Network: Towards Unity for Health, a global consortium of health professions schools.

Datta Meghe Institute of Medical Sciences, Wardha established the Department of Health Professional Education in 2009 and offers an Advanced Course in Medical Education recognized by MCI and 30% of the faculty of Datta Meghe Institute of Medical Sciences have been trained in Advanced Courses in Medical Education. DMIMS is also a MCI Regional Centre and has trained more than 1000 medical teachers from more than 15 medical colleges.  The institute also has six FAIMER faculty and three National Faculty for Advanced Courses in Medical Education recognized by MCI. South East Asia Regional Association for Medical Education of the World Federation for Medical Education is also supporting in this endeavor by promoting partnerships and linkages with various local and regional associations to collaborate for medical education under the umbrella of World Federation for Medical Education. 

## Discussion

Though several initiatives are being undertaken by various agencies to build the pedagogy skills of medical teachers, the flipside is that the number of workshops, trainings and courses are too meager to address the huge need. In near future, in addition to the existing medical colleges, HLEG on UHC has proposed establishing new 187 medical colleges by 2022(15). These medical colleges will be having higher intake capacity and therefore will require more medical teachers to be trained through FDPs.

Although the MCI Basic Course has been made compulsory for medical teachers to the level of professor and teacher administrators, it is also being recommended that the Medical Education Units in all medical colleges should train all existing medical teachers in Basic Course and conduct the Basic Course for newly inducted faculty twice a year(2). MCI Undergraduate Working Group Document in July 2010 shed light on the training capacity analysis and envisaged that 40000 medical teachers are needed to be trained by 4000 trained teachers (16). But the assessment of quality of these workshops only on the basis of pretest and post-test will not be sufficient. Evaluation needs to be conducted in terms of the competencies acquired by the medical teachers who have undergone these trainings. The number of trainings conducted and the number of participants trained cannot be the only parameter for determining the teaching skills acquired by the medical faculty(15). We also need to look upon scaling up without comprising on the quality of FDPs. Distance learning courses focusing on educational leadership and pedagogy for medical teachers can be an option to reach wider audience.

Nodal faculty development centers, Medical Education Units and Centers of Regional FAIMER Institutes in India have been able to do the capacity building and ultimately helping to increase the ability of systems to function on their own to meet local needs (12). MCI has made the basic course of medical education mandatory for appointment as a teacher in medical school. All the medical colleges have a designated medical education unit, and many have dedicated staff and facilities in medical education. Regular curricular reviews and revisions, improvement of teaching and learning activities and assessments and staff development are now routinely undertaken(5). However, there is an urgent need to formulate a National Faculty Development strategy for medical teachers for catering to the desired objectives(9-11).

A big lacunae at present is in terms of creating and developing an educational leadership. Programs need to be designed to educate policy makers and update them about recent advances in medical education worldwide. These include engaging people’s moral purposes, building capacity to generate forces for change, understanding the change process, developing the learning culture and the culture of evaluation and fostering development at all possible levels. 

## Conclusion

This report highlights the need for strengthening faculty development as a vehicle for ensuring quality in medical education. There is evidence, in most countries, educators of health professionals are insufficiently prepared as teachers and trainers, even though their clinical knowledge and skills may be good. The success of educational reforms ultimately lies with the individual instructors and their capacity, individually and collectively to execute and implement some novel ways in teaching and training the future cadre of doctors and more importantly with India progressively becoming a new global hub of education. Faculty development efforts should empower the medical teachers and keep the passion in teaching going so that the lifelong learning never ceases. 
